# Predicting attachment style from EEG data on the Flanker task

**DOI:** 10.3389/fnhum.2025.1651943

**Published:** 2025-09-05

**Authors:** Dor Mizrahi, Inon Zuckerman, Ilan Laufer

**Affiliations:** Department of Industrial Engineering and Management, Ariel University, Ariel, Israel

**Keywords:** EEG data, attachment theory, Flanker task, predictive modeling, emotional bonds

## Abstract

Bowlby's attachment theory describes the differences that people exhibit in the way they form emotional bonds with others. The dimensional measure of attachment describes it by the magnitude of anxiety and an avoidance dimension, which are currently measured using a self-report questionnaire. Recent advances in neurophysiological methods have started exploring the neural underpinnings of attachment styles. Nonetheless, a conspicuous gap remains: the underexplored realm of predictive models for predicting attachment styles based on objective physiological data. With that in mind, we have constructed a model for inferring individual attachment profiles, based solely on their brain signals recorded using an electroencephalogram (EEG). For that aim, we recorded EEG data of 27 participants engaged in the Flanker task and receiving either positive or negative feedback following each trial. We then utilized the recently developed ROCKET algorithm (RandOm Convolutional KErnel Transform) to automatically extract 20,000 time-series features from the EEG data. Next, we applied a Principal Component Analysis (PCA) and reduced the number of features to 87 individual components that were used to construct regression models predicting participants' anxiety and avoidance scores, as measured by the ECR-R questionnaire. Our results show, for the first time, that individual attachment profiles can be inferred from EEG data, allowing *post hoc* categorization into the four canonical attachment styles. This offers two key contributions: first, it provides an objective alternative to traditional self-report questionnaires, helping reduce subjectivity bias in attachment assessment. Second, it highlights the value of using automatically generated features over the limited set of hand-crafted features typically found in the literature.

## 1 Introduction

Bowlby's Attachment theory posits that people exhibit differences in how they form emotional bonds with another person (Fearon and Roisman, 2017). The first distinction in attachment styles is either a *secure* or an *insecure* attachment style. A *securely attached* individual shows mutual trust and support, is emotionally stable in conflict situations, and can set healthy boundaries. Individuals with insecure attachment styles are roughly divided into having an *anxious* or an *avoidance* attachment style. *Anxious* individuals have an intense fear of abandonment and a need for constant validation. They are dependent on their partner for self-worth. *Avoidant* individuals, as the name suggests, avoid intimacy and vulnerability, have commitment issues, and have guarded and closed-off hearts. Some individuals exhibit both a high degree of anxiety and a high degree of avoidance and are known as having a *fearful-avoidant* (or *disorganized)* attachment. They have a strong fear of rejection, difficulty trusting and relying on a partner, and low self-esteem.

While the attachment style does have a genetic ingredient which seems to contribute to around 40% of the variability of anxiety and avoidance dimensions (Erkoreka et al., 2021), it is formed mainly by the way the baby is taken care of by his primary caretaker in its early years, mostly between birth and until age three (Fearon and Roisman, 2017). In addition, the attachment style is primarily stable throughout the years. It profoundly impacts human behavior, particularly the significant emotional bonds in his adult life and his neurophysiological responses (Jones et al., 2018). Previous research showed varying degrees of statistical distributions of the attachment styles in the population; however, most studies show that roughly 50% of the population exhibits a secure attachment style, around 20% exhibit an anxious and avoidant attachment style, and around 10% are those with a fearful avoidant attachment style (Waters et al., 2000; Fearon and Roisman, 2017).

There are currently two main methods for measuring adult attachment style: a self-report questionnaire and a narrative (Gander and Buchheim, 2015). There are many self-report questionnaires with different attributes; some provide a discrete classification of one of the four attachment styles, while others measure the degree to which each attachment dimension (anxiety or avoidance) is present (Ravitz et al., 2010). For example, the ECR-R (Experiences in Close Relationships—Revisited) questionnaire, which includes 36 items, is a reliable and valid self-report questionnaire that quantifies the anxiety and avoidance dimensions to a value in the range of 1 (lowest) to 7 (highest) (Sibley et al., 2005). Thus, for example, an individual who scored 1.5 on the anxiety dimension and 4.5 on the avoidance dimension will be denoted as having an avoidant attachment style. An individual who scored low on both dimensions will be classified as having a secure attachment style.

In addition to psychological studies, there has been a growing body of research exploring the physiological aspects of attachment styles. Converging evidence from a recent systematic review shows that insecure attachment is consistently associated with impaired emotion regulation across autonomic, biochemical, EEG, and behavioral indices, underscoring the neurophysiological basis of attachment (Eilert and Buchheim, 2023). Researchers have examined various physiological responses, such as cardiovascular activity and galvanic skin conductance, while others have focused on adrenocortical activity (see Gander and Buchheim, 2015 for a review). These studies suggest correlations between attachment behaviors and physiological reactions. Complementing EEG findings, resting-state fMRI work has linked individual differences in attachment anxiety and avoidance to distinct intrinsic brain dynamics, both amplitude of low-frequency, fluctuations and functional connectivity patterns in networks including the posterior cingulate and fusiform regions (Deng et al., 2021). More recently, advancements in electroencephalogram (EEG) studies have provided insights into the neural correlates of attachment. For example, (Verbeke et al., 2014) demonstrated that social environments significantly influence cortical activity, especially among individuals with anxious attachment, showing heightened alpha, beta, and theta band activity in social settings. This discovery offers potential explanations for their social behavior and interaction patterns. Similarly, (Sloan et al., 2007) identified a link between attachment anxiety and the presence of alpha waves during sleep, and (Rognoni et al., 2008) highlighted the relationship between adult attachment styles and EEG frontal asymmetry, which contributes to understanding emotional regulation. Furthermore, research on EEG event-related potentials (ERPs) (Zuckerman et al., 2023b; Laufer et al., 2024) has illuminated how attachment styles affect defensive responses, notably in moderating the attachment system through P200 and P400 ERPs during the Flanker task. In addition, network-level EEG analyses show reduced global efficiency following attachment-memory retrieval in unresolved/disorganized attachment, further supporting a neurophysiological substrate for attachment representations (Massullo et al., 2022).

While these findings have advanced the field, there remains a notable gap in the development of predictive models that can forecast attachment styles based on physiological data. Physiological measurements offer an advantage over self-report questionnaires by providing more objective and accurate data, as they bypass the potential biases associated with self-reports. Specifically, EEG brain signals—using time-domain and frequency-domain analyses and transformations such as wavelets and various Fourier transforms together with complex mathematical representations that capture essential characteristics and relationships of data from the real world (i.e., signals embeddings) (Li et al., 2018; Alhalaseh and Alasasfeh, 2020; Jaswal and Dhingra, 2023; Mizrahi et al., 2023; Vempati and Sharma, 2023) creates a rich set of features for building models capable of predicting an individual's attachment style.

Considering the abovementioned gap in knowledge, our research goal was to construct a model for predicting the attachment of adult individuals, their anxiety, and avoidance values based solely on their EEG signals. To align with this goal, we focused specifically on the neural activity that occurs immediately after participants received performance feedback (success or failure) in the Flanker task, as this period is when the emotional response to the outcome is most likely to be evoked. By targeting post-feedback epochs rather than stimulus selection periods, we aimed to capture attachment-related differences in affective processing under controlled task conditions. For that aim, we recorded EEG data of participants who were engaged in Erikson's Flanker task (Brunetti et al., 2019) that was originally introduced to study cognitive control and attentional processes with no direct connection to attachment styles. Our rationale for selecting the Flanker task, despite its primary use in cognitive control research, is threefold. First, prior work shows attachment-related differences in EEG during cognitive tasks (e.g., Zuckerman et al., 2023a) and the feasibility of EEG-based attachment prediction more broadly (Laufer et al., 2024). Second, the Flanker task uses neutral, highly controlled stimuli and a fixed target location—minimizing variability from emotional or semantic interpretation and yielding consistent neural responses across participants (Eriksen and Eriksen, 1974; Fan et al., 2002). Third, it is simple and robust for eliciting well-characterized ERP markers of control (e.g., N2, ERN/Pe), supporting detection of subtle between-group differences without introducing overt affective content (Folstein and Van Petten, 2008; Lin et al., 2020).

The EEG data was preprocessed and analyzed using the recently developed ROCKET algorithm (RandOm Convolutional KErnel Transform) (Dempster et al., 2020) that created a comprehensive set of 20,000 automatically generated features from the EEG data. In other words, while there are known features in the literature that can be used to some degree in differentiation between attachment classes [for example, P200 and P400 ERP that were utilized in Laufer et al. (2024) or enhanced Alpha and Beta bands as was reported in Verbeke et al. (2014)], the novelty of the ROCKET algorithm is that by using convolutional networks, it automatically constructs a large number of features that are statistical in nature. Thus, those features lack a specific interpretation in the context of brain neuroscience, however, as our results show, they can be extremely useful for the construction of a machine learning prediction model.

The large set of features extracted through the ROCKET algorithm was later reduced using Principal Component Analysis (PCA) to distill the data into its most informative 87 principal components to ensure the clarity and relevance of our model's inputs. On these 87 features, we run the CatBoost machine learning algorithm, renowned for its proficiency with categorical data and regression tasks (Hancock and Khoshgoftaar, 2020), to predict each of the four established attachment styles—secure, avoidant, anxious, and fearful-avoidant (Mikulincer and Shaver, 2005).

Our results are two-fold: First and foremost, we present a regression model that predicts the anxiety and avoidance dimensions of the participant's attachment style by analyzing a *single* epoch of the Flanker task. Second, we show a dependency between the anxiety and avoidance dimensions by comparing the error rates of two regression models: multi-target regression and two single-value regressions. The results of the paper show, for the first time, that attachment style can be predicted using EEG data. Prediction using EEG provides an alternative way of measuring attachment, which reduces the subjectivity bias of the standard self-report questionnaires or narrated interview methods.

## 2 Experimental design

The study consisted of two primary phases. First, 96 participants filled in the ECR-R questionnaires (with 36 items), and then 27 participants from the first group were invited to the EEG laboratory for the second phase, where they engaged in the Flanker task while their EEGs were recorded. The EEG sample consisted of 27 participants (16 women and 11 men) with a mean age of 23.8 years (men: 24.9 ± 2.6 years; women: 23.06 ± 1.8 years). All participants were right-handed and enrolled as undergraduate engineering students, resulting in a relatively homogeneous sample in terms of age, education level, and handedness. While age and gender were not included as covariates in the analyses, the limited variability in these characteristics reduces the likelihood of substantial confounding effects. The experiment received approval from the institution's Institutional Review Board (IRB) committee, and all participants signed a formal agreement form before participating.

### 2.1 Data collection

**Figure 1:**
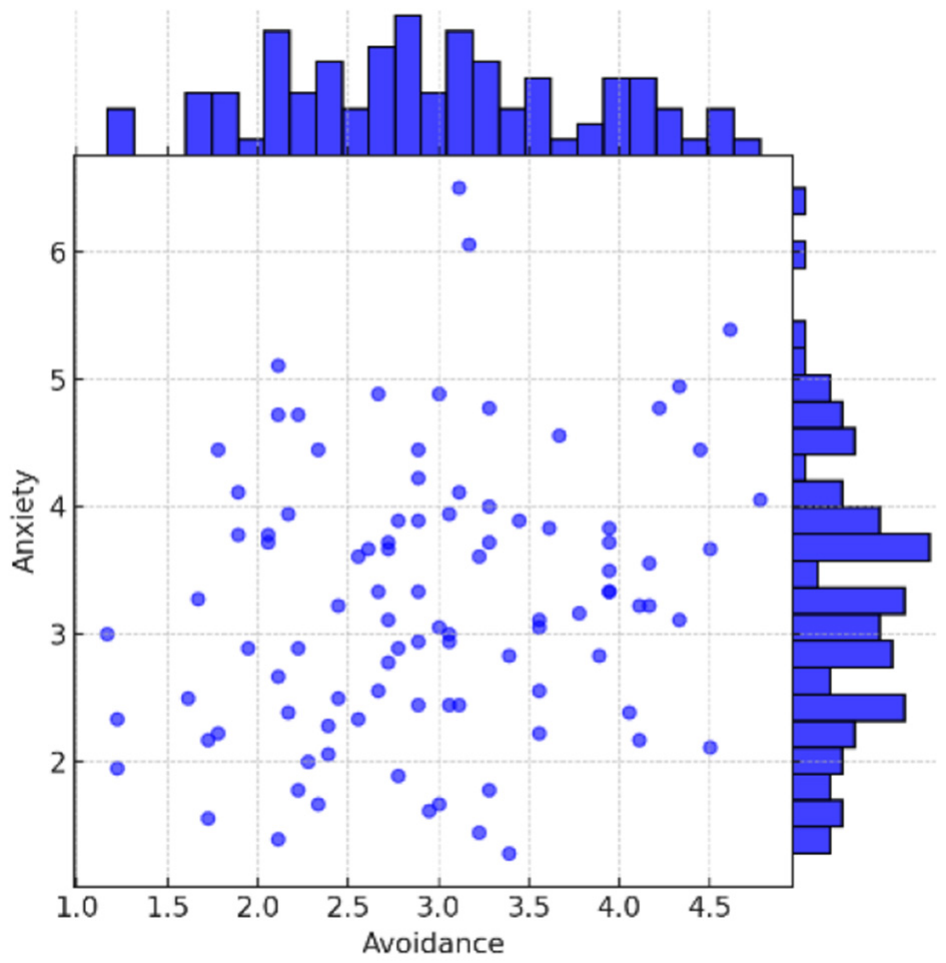
Attachment results of the ECR-R questionnaire.

The ECR-R questionnaire outputs two values for each participant, anxiety and avoidant, on a scale of 1 to 7. Figure 1 shows the distribution of the ECR-R results. The x-axis shows the avoidance dimension value of the participant, and the y-axis shows her anxiety dimension value. The values range from 1 to 7, where the highest value indicates an increase in both insecurity dimensions. Secure attachment can be seen in participants with low values in both dimensions, and fearful avoidance is seen in those with high values in both dimensions.

**Figure 2:**
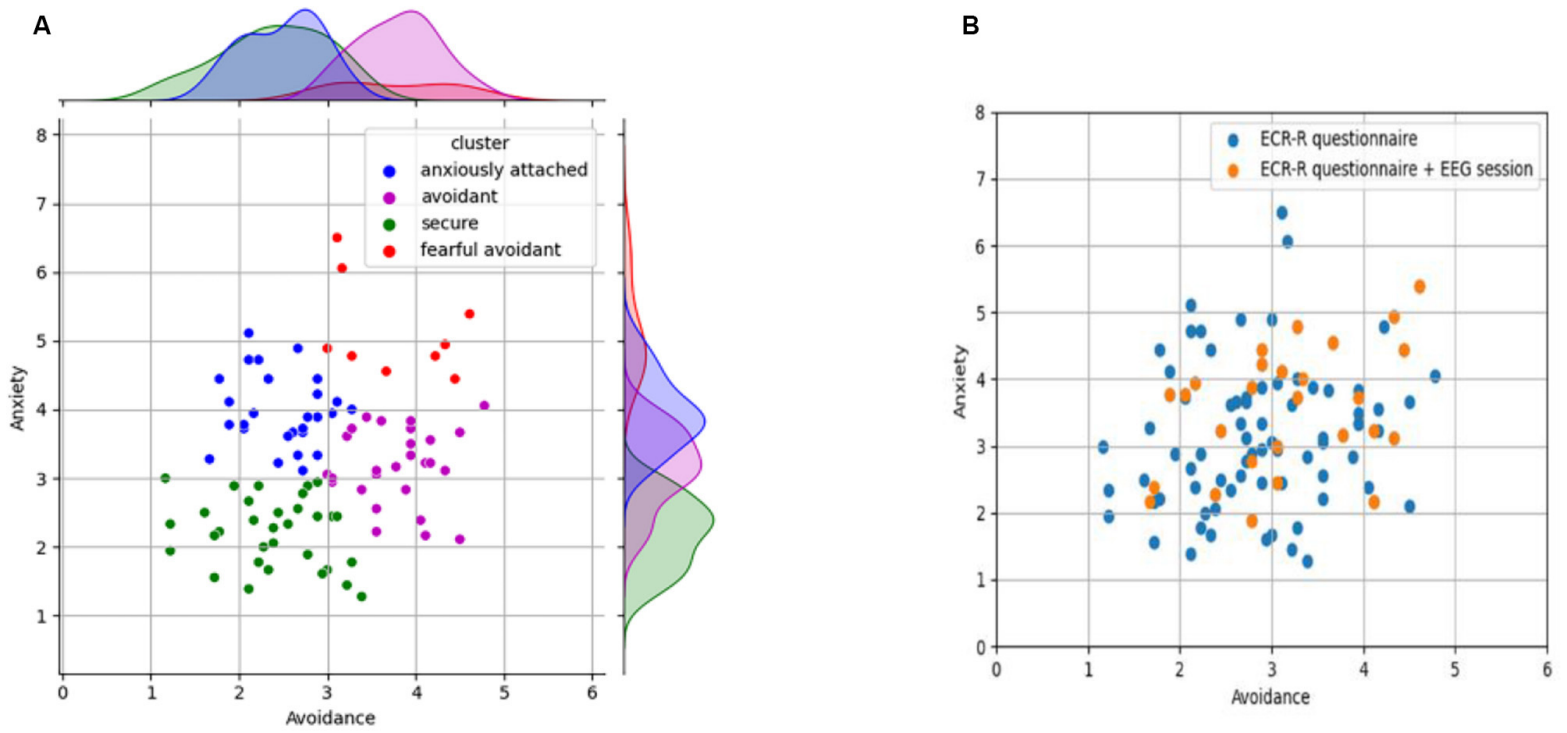
**(A)** Grouped attachment outcomes based on the ECR-R questionnaire (K = 4) **(B)** the sample of individuals who participated in the 2^nd^ stage of the experiment, the EEG session.

Next, we employed the k-means clustering algorithm to autonomously determine the appropriate number of clusters within the provided dataset. To identify the optimal number of clusters, we applied the Elbow method (Syakur et al., 2018), which, in line with the established literature regarding the existence of four distinct attachment classes, selected k = 4 as its optimal value (see Figure 2A). The different groups were created by the k-means algorithm and will be utilized to select participants for the 2nd stage of the experiments. The exact values of the boundaries are not crucial to our research, which will predict the continuous anxiety/avoidance values and not group membership.

In the second stage, we issued invitations to participate in the EEG part of the experiment. Each session was 1 h long, and participants were paid for their efforts. We employed a proportional allocation method to ensure a representative distribution across attachment clusters. This method kept the proportions consistent with the relative sizes of each attachment cluster. The secure group consisted of six participants; nine were anxiously attached, seven were avoidants, and five participants had a fearful avoidant attachment. Figure 2B shows the 27 individuals who participated in the EEG recording part of the experiment. You can see that the orange dots provide a uniform sample of the population from the first part of the experiment.

During this phase, the participants engaged in Eriksen's Flanker task (Brunetti et al., 2019). The Eriksen Flanker task (developed in 1974) is a widely used experimental paradigm in cognitive psychology and neuroscience to study cognitive control and attentional processes. The task involves participants responding to a central target stimulus flanked by distracting stimuli. It has proven valuable in investigating various executive functions and cognitive control aspects in healthy individuals and clinical populations. As explained below, our main interest is not in the cognitive process during the selection but in the evoked emotional response after a successful or unsuccessful decision. Accordingly, the EEG analysis in this study was designed to focus on the time period immediately following the feedback screen, which is when the emotional response to the trial outcome is expected to occur. All epochs used for feature extraction were time-locked to the onset of feedback (correct in green or incorrect in red), enabling us to capture neural activity associated with processing success or failure, rather than the cognitive demands of stimulus selection.

**Figure 3:**
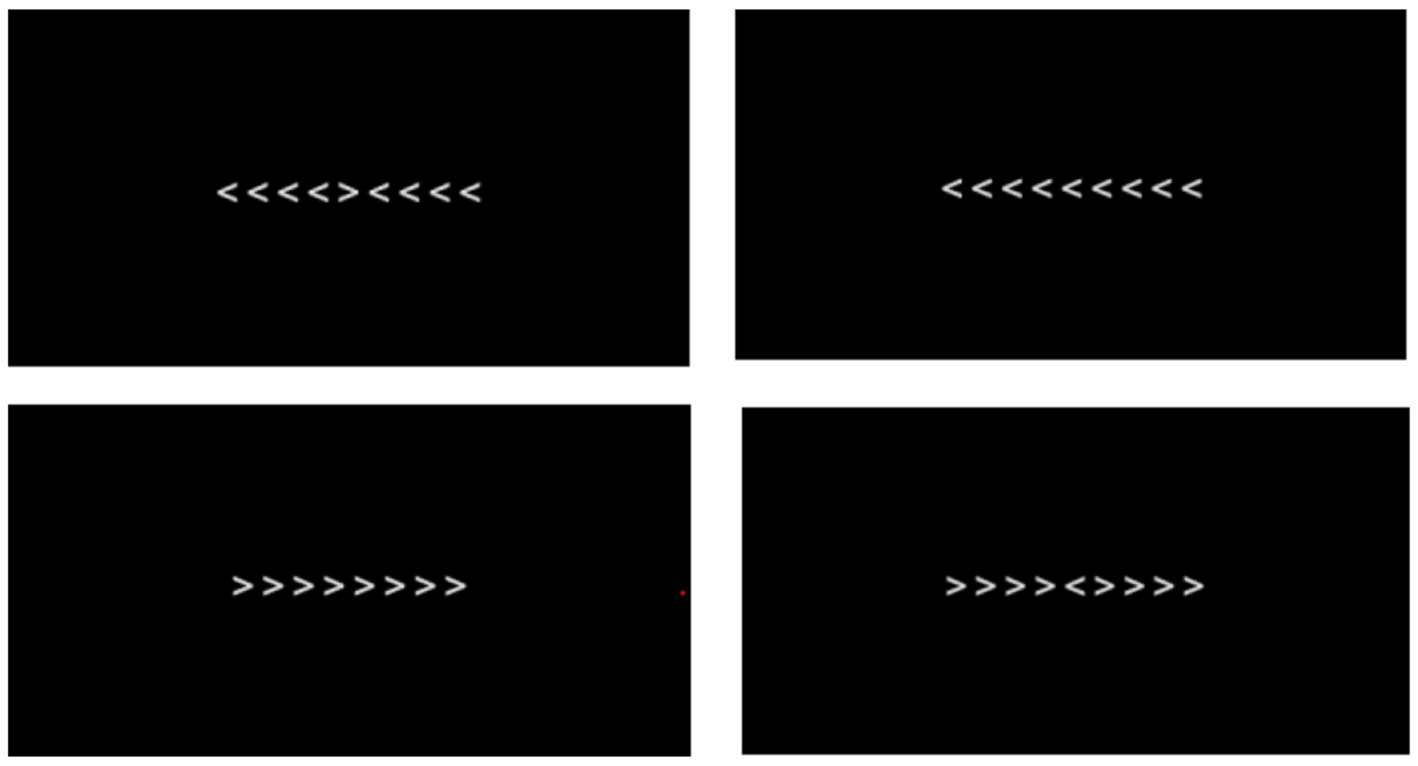
The Flanker task.

In our adaptation of the Flanker task, arrow symbols were used, and participants were presented with one of four possible arrow configurations (see Figure 3) for a duration of 1 s:

1. Congruent right (→→→)
2. Congruent left (←←←)
3. Incongruent right target (← → ←)
4. Incongruent left target (→←→)

The middle arrow served as the target, and the flanking arrows served as distractors. On each trial, one of these four configurations was selected randomly with equal probability, ensuring that across each block approximately half the trials were congruent and half incongruent.

The task consisted of three blocks of 20 trials each. In the first and third blocks, participants pressed the key corresponding to the direction of the central arrow (congruent keypress–arrow mapping). In the second block, participants pressed the opposite key (incongruent keypress–arrow mapping). This design ensured that participants had to actively engage cognitive control processes in the second block rather than relying on an automatic response strategy from the first block. Congruent and incongruent flanker arrangements were presented in all blocks to maintain interference effects throughout the task.

In our analysis, interference was quantified by comparing performance (reaction time and accuracy) between congruent and incongruent trials, enabling us to evaluate how distractor conflict influenced task execution across different keypress–arrow mapping conditions. After each trial, a feedback screen was presented for 1 second, showing “correct” in green or “incorrect” in red based on performance. Before the main task, all participants completed a short training session to become familiar with the procedure.

**Figure 4:**
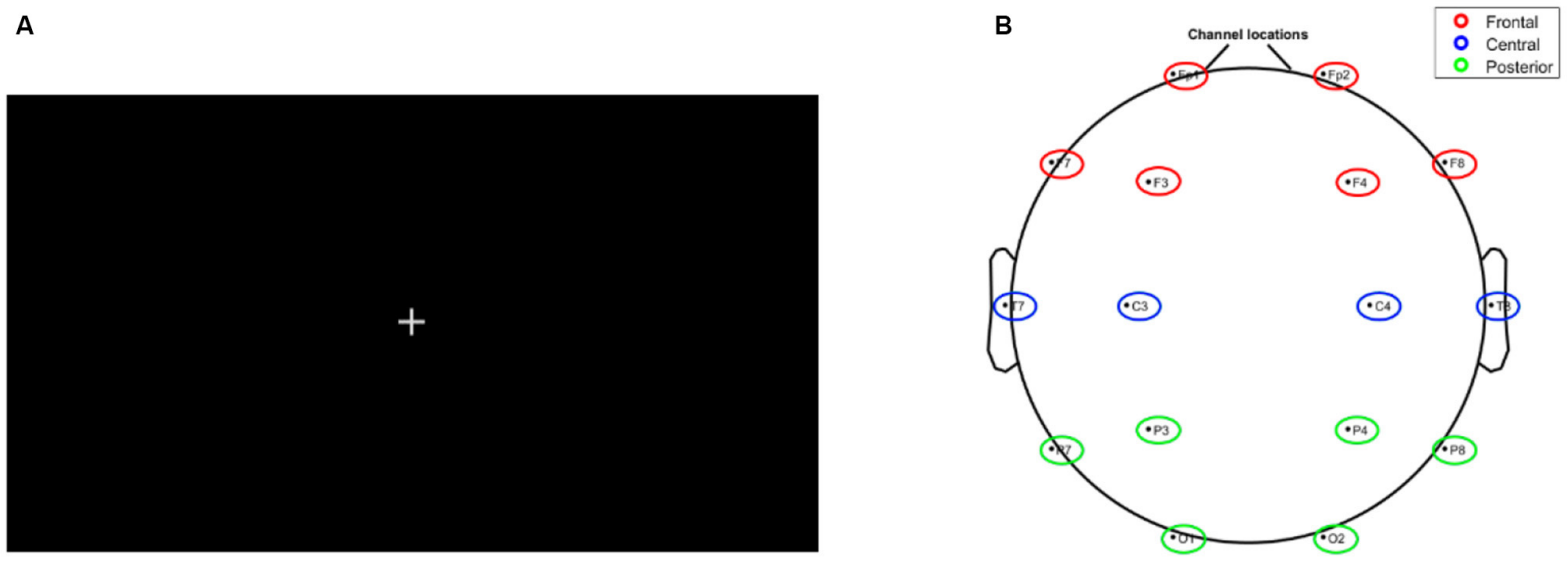
**(A)** Fixation cross **(B)** the 10–20 position of the electrodes.

A fixation cross (Figure 4A) was displayed between trails, and its duration varied randomly between 0.5 and 1.5 s.

EEG signals were recorded using a 16-channel active EEG amplifier (USBAMP, by g.tec, Austria) operating at a sampling frequency of 512 Hz, adhering to the 10–20 international system using 512 Hz (Figure 4B). Electrode impedance was maintained below 5 Kohm throughout the experiment, and data analysis focused on six frontal and prefrontal electrodes (Fp1, F7, Fp2, F8, F3, and F7).

## 3 Data analysis

### 3.1 Preprocessing step

EEG signals were recorded at a sampling rate of 512 Hz using a 16-channel cap arranged according to the international 10–20 system, with the Cz electrode serving as the reference. The raw EEG data underwent bandpass FIR filtering between 1–32 Hz to remove slow drifts and high-frequency noise, followed by a 50 Hz notch FIR filter to attenuate power-line interference. Signals were then re-referenced to the average of all electrodes to reduce spatial bias.

Artifact removal was performed using Independent Component Analysis (ICA), which allowed the separation of neural and non-neural sources. Components corresponding to ocular (blinks, saccades) and muscle artifacts were visually identified and removed. The cleaned continuous EEG data were then segmented into 1-second epochs, time-locked to stimulus onset, aligning with the Flanker task slide duration. Time-locking to stimulus onset ensured consistent alignment of neural responses across trials and participants, facilitating direct comparison of EEG features related to task performance.

**Figure 5:**
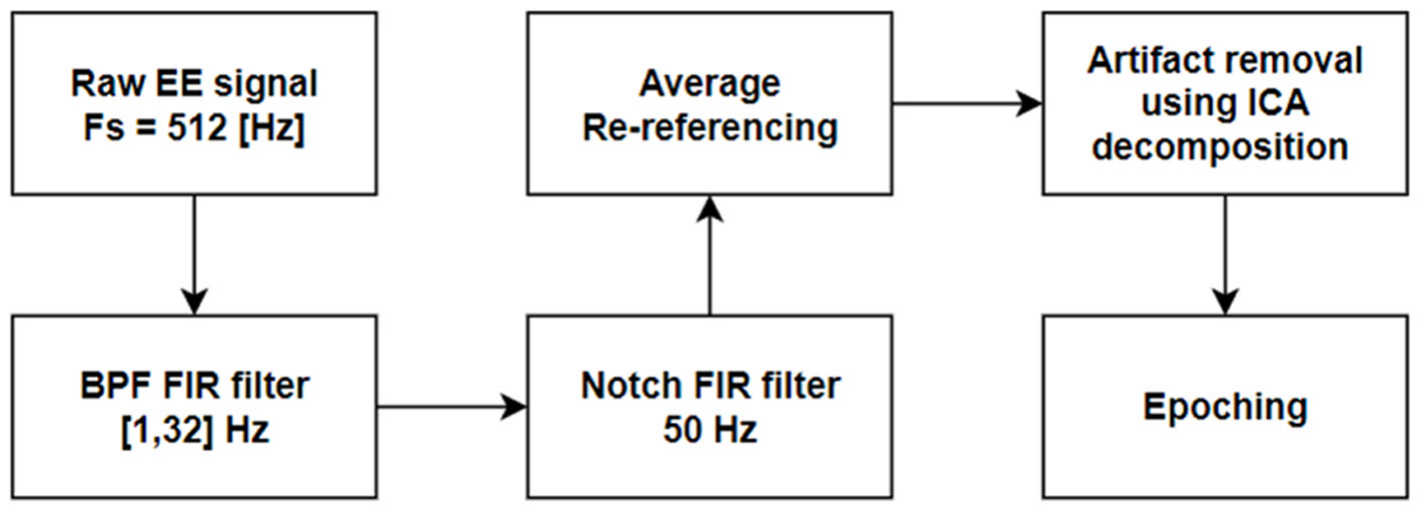
EEG preprocessing scheme.

The complete preprocessing pipeline is illustrated in Figure 5.

### 3.2 Feature extraction

After acquiring and preprocessing the EEG data, we segmented the continuous recordings into epochs time-locked to the onset of visual feedback, indicating success or failure in the Flanker task. Each epoch lasted for 1,000 ms after feedback presentation and included all trials, irrespective of whether the feedback was positive or negative. Feedback type was not separated in this analysis, as the aim of the present study was to model general post-feedback neural responses rather than to examine differences between feedback conditions. This pooling across feedback types was a deliberate design choice, reflecting the main aim of the study—to predict attachment style from general post-feedback neural activity rather than to examine specific differences between success and failure conditions. Pooling also increased the total number of trials available for analysis, thereby reducing the impact of random variation or noise due to small sample sizes in each feedback condition. In addition, this approach ensured more balanced datasets, which improves the performance of the classification algorithms employed in this study.

We utilized the ROCKET algorithm (Dempster et al., 2020) for feature extraction. ROCKET is specifically designed to handle time series data rapidly. It uses a diverse set of randomly selected convolutional kernels to transform raw EEG time series data into a high-dimensional feature space. This approach allows ROCKET to uncover significant patterns and structures within the data, regardless of size or complexity. From the EEG epochs, ROCKET generated an extensive array of 20,000 features.

Given the relatively small number of trials per participant and the sensitivity of the ROCKET algorithm to extreme values, we implemented measures to minimize the influence of outliers before feature generation. Trials with reaction times exceeding three standard deviations from the participant's mean were excluded to remove atypical behavioral responses likely caused by distraction or technical issues. In addition, all features were standardized to z-scores within participants before applying ROCKET, ensuring that differences in signal amplitude or scale did not disproportionately affect the convolutional kernel outputs. These steps reduced the risk of distortion from extreme values while preserving meaningful neural variability relevant to attachment-related processes.

To manage the complexity and high dimensionality of this feature set, we applied Principal Component Analysis (PCA). PCA reduces the dimensionality of data while preserving as much variance as possible by transforming the original features into a new set of orthogonal components, thereby identifying the most informative aspects of the data.

In our analysis, PCA reduced the 20,000 features to 87 principal components, selected based on their ability to retain over 90% of the original variance. This high retention rate ensures that the condensed feature set retains the essential information for our predictive modeling. By focusing on these principal components, we minimized the risk of overfitting and improved the generalizability of our regression models, which supports the accuracy of predicting attachment styles from EEG data.

### 3.3 Constructing the regression models

**Figure 6:**
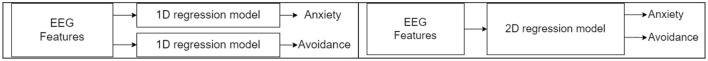
The prediction model structure. The left panel shows separate regression models for predicting avoidance and anxiety. The right panel illustrates a multi-target model that predicts both simultaneously, leveraging correlations to improve accuracy. All models were evaluated using a stratified K-fold cross-validation procedure (K = 3), ensuring equal representation of all four attachment groups in each fold and computing performance metrics exclusively on held-out test sets.

Figure 6 schematically depicts the structure of our predictive models, both of which use 87 principal components derived from the original 20,000 features extracted from the EEG data. The left panel shows the separate multiple linear regression models, where two independent models are trained: one for predicting the avoidance value and one for predicting the anxiety value. Each model uses the 87 features independently and does not consider the potential relationship between anxiety and avoidance.

The right panel presents the single multi-target linear regression model, designed to simultaneously predict anxiety and avoidance values from the 87 features. This model leverages the potential correlations between the two target variables, improving its predictive capability. Specifically, if there is a correlation between anxiety and avoidance, the multi-target model can use this relationship to make more accurate predictions. For example, if higher anxiety often correlates with higher avoidance, the model can adjust its predictions to reflect this relationship, enhancing its overall performance.

The need to construct and select between the two models arose for two reasons: First, we hypothesized from the psychological literature that a covariance exists between the two variables, but we could not approximate its magnitude. Second, using a larger model, such as in the multi-target case, might result in overfitting, as our data was relatively small for an 87-features prediction problem. By simultaneously predicting both values, the multi-target model can improve accuracy by leveraging the interrelated nature of anxiety and avoidance. Conversely, if anxiety and avoidance are largely independent, separate multiple linear regression models might perform just as well or better. These models treat each target variable independently, which can be beneficial if no significant interaction exists between them.

We utilized the supervised learning paradigm for the construction of the prediction model. However, while a wide range of supervised learning algorithms exist (decision trees, artificial neural networks, support vector machines, and others), each with its strengths and weaknesses, no single learning algorithm works best on all supervised learning problems. For our cause, mainly due to our relatively small data set, we have decided to use a decision-learning tree ensemble called CatBoost (implemented in Python) to construct the models for the computation. CatBoost, a tree-based ensemble model (Hancock and Khoshgoftaar, 2020), offers fast computation for dual regression tasks, allowing for the simultaneous prediction of anxiety and avoidance. CatBoost utilizes several cost functions, and we applied the Root Mean Square Error (RMSE), which measures the average error between observed and predicted values.

To ensure the validity and generalizability of our models, we employed a stratified K-fold cross-validation procedure with K = 3. In each fold, the data were divided into training and test sets such that all four attachment groups (secure, avoidant, anxious, and fearful-avoidant) were equally represented in both sets, thus preventing class imbalance from biasing the results. Model training was performed exclusively on the training set for each fold, and performance metrics (RMSE) were computed only on the held-out test set. This process was repeated for all folds, and the reported results reflect the aggregated performance across the three test folds. This approach mitigates overfitting and provides a more reliable estimate of out-of-sample predictive performance, in line with best practices for small to moderate datasets.

The performance comparison between these models is detailed in Table 1 (in the Results section), which provides the error distances for each model. The multi-target regression model shows superior performance with lower mean error distances, highlighting the benefit of accounting for the interdependence between anxiety and avoidance in a single predictive model. By accounting for this interdependence, the multi-target model can more accurately capture the underlying patterns in the data, leading to better predictive performance. This approach allows the model to use the shared information between anxiety and avoidance, resulting in predictions that are not only more precise but also more reflective of the true nature of the psychological constructs being studied (Mizrahi et al., 2024).

**Table 1:** Mean ± SD reaction time (RT) in milliseconds and accuracy for each attachment class.

**Attachment class**	**N**	**RT mean ±SD (ms)**	**Accuracy mean ±SD**
Secure	6	569.8 ± 54.6	0.893 ± 0.095
Avoidant	7	577.8 ± 38.3	0.953 ± 0.046
Anxious	9	592.3 ± 41.2	0.922 ± 0.055
Fearful-avoidant	5	546.5 ± 61.9	0.878 ± 0.118

N = number of participants per class.

## 4 Results

### 4.1 Behavioral results

Across the full dataset (*N* = 27 participants; 1,620 trials), the mean reaction time (RT) at the trial level was 571.4 ± 127.3 ms. Aggregating to the participant level, the mean of per-participant RTs was 571.4 ± 50.2 ms. Accuracy was high overall: 0.910 at the trial level; the mean of per-participant accuracies was 0.910 ± 0.090.

Associations between attachment dimensions and performance were assessed at the participant level. Anxiety showed no reliable association with mean RT (*r* = −0.012, *p* = 0.953) or accuracy (*r* = 0.285, *p* = 0.150). Avoidance showed a small, non-significant negative association with mean RT (*r* = −0.176, *p* = 0.380) and a moderate positive association with accuracy (*r* = 0.538, *p* = 0.004). Taken together, these results indicate that individual differences in behavioral performance did not systematically covary with anxiety and only modestly covaried with avoidance (higher avoidance associated with higher accuracy), suggesting that the EEG-based effects reported below are unlikely to be explained by broad performance differences.

A descriptive summary of mean RT and accuracy by attachment class is presented in Table 1.

In addition, in an exploratory analysis, age and gender were tested as potential confounding variables. Neither variable was found to be significantly associated with RT, accuracy, or the attachment dimensions, suggesting that demographic factors did not exert a notable influence on the observed results.

### 4.2 Comparing the two regression models

A feature selection process was applied to improve the performance and reliability of both the separate multiple regression models and the multi-target regression model. Using PCA, the original 20,000 features were reduced to 87 principal components. This dimensionality reduction helps prevent overfitting, which occurs when a model captures noise rather than meaningful patterns in the data. By retaining over 90% of the original variance, PCA preserved the most significant and informative features. This approach simplifies the models, reducing complexity and enhancing their generalization ability to new, unseen data. Consequently, the regression models are less likely to overfit and are better equipped to make accurate and reliable predictions.

Table 2 depicts the mean error distance of the regression outputs to the centroid of the attachment style. Recall that the values of both anxiety and avoidants are in the range of 1 to 7 in the ECR-R questionnaire, and we can see that the values of the error distance are relatively small in both cases. However, it is easy to see that the mean error values of the multi-target regression in all four attachment classes are *smaller* than in the two separate multiple regressions. The most minor improvement, 3.54%, was in the Anxious class, and the most significant difference, 27.91%, was in the Fearful-avoidant class. The Secure and Avoidant classes improved 11.72% and 13.85% respectively.

**Table 2:** Comparison of both model's mean error distances.

**Single multi-target regression model Mean error distance**	**Multiple 1D regression models Mean error distance**	**Attachment style**
0.371969	0.415561	Secure
0.729351	0.830325	Avoidant
0.791785	0.819838	Anxious
0.414988	0.530712	Fearful-Avoidant

As stated earlier, we can now see through these results that there is some dependency between the Anxiety and the Avoidance values. Therefore, we continued our analysis using the multi-target regression model, which proved to be a better fit for the problem.

### 4.3 Exploring the mean error for each attachment class

**Figure 7:**
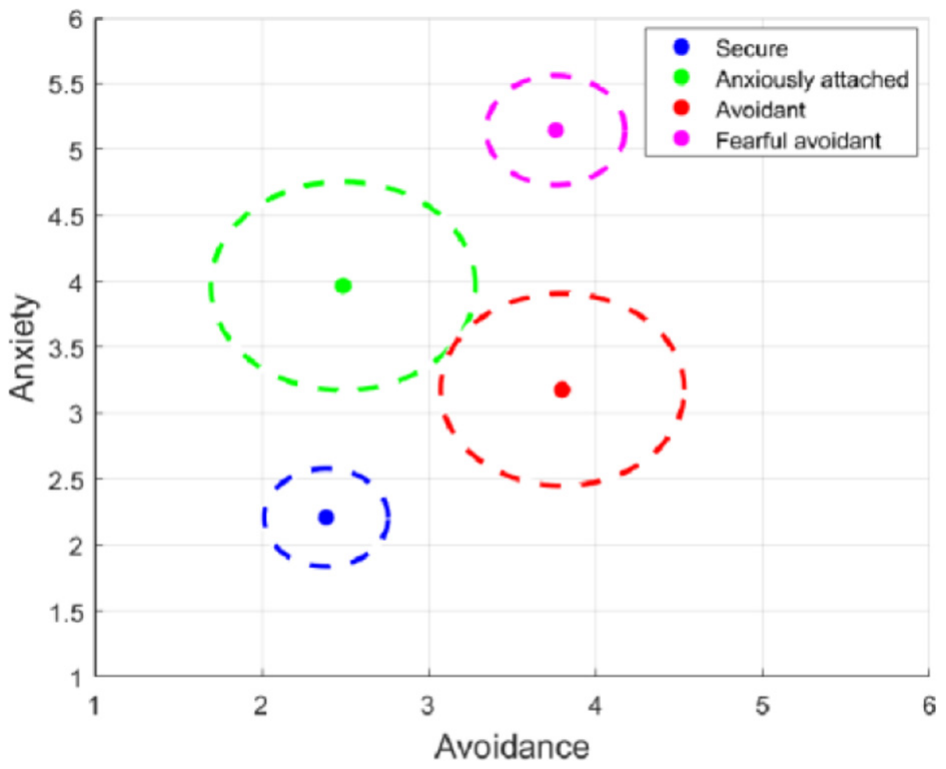
Visualization of the mean error for each attachment class.

Figure 7 presents the average centroid locations calculated using k-means clustering within a two-dimensional space representing Avoidance and Anxiety. The circles in the graph indicate the average distance of data points within each cluster, serving as a proxy for prediction error. The size of these circles reflects the regression model's accuracy in classifying each attachment style. In other words, the radius corresponds to the mean error of each cluster.

Interpreting the visual information in Figure 7 and considering the radius of each circle, which signifies data compactness or error size, we can draw the following conclusions:

1. Secure cluster (blue): The blue circle, representing the Secure attachment style, is tightly clustered with a small radius. This suggests that individuals classified under the Secure attachment style tend to have minimal variability in their Anxiety and Avoidance scores. Consequently, the model's prediction error for this cluster is expected to be low (mean error of 0.37), indicating high precision in identifying individuals with Secure attachment characteristics.
2. Anxious cluster (green): The green circle has a larger area than the blue one, indicating more variability among individuals within the Anxiously Attached category. This implies that while the central tendency for Anxiously attached individuals is characterized by higher Anxiety and lower Avoidance, there is less consistency in the model's predictions for this group, as evidenced by the larger circle radius (mean error of 0.79).
3. Avoidant cluster (red): The red circle is slightly smaller than the green one, suggesting a comparable prediction error or variability level for the Avoidant attachment style (mean error of 0.73). Individuals in this cluster exhibit higher Avoidance but lower Anxiety. The spread of this cluster also indicates less consistency in the model's predictions for this group.
4. Fearful Avoidant cluster (magenta): The magenta circle is smaller than the green and red circles but slightly larger than the blue one (mean error of 0.41). This cluster comprises individuals with high levels of both Anxiety and Avoidance. Nevertheless, the relatively tight cluster suggests that the Fearful Avoidant attachment style exhibits predictive accuracy comparable to the Secure group, with somewhat less variation in the model's classification of these individuals than those with Anxious or Avoidant attachment styles.

In summary, the secure cluster stands out with the most compact grouping, emphasizing the model's exceptional performance in predicting this attachment style. On the other hand, the anxious and avoidant clusters, represented by larger circles, display varying degrees of prediction error and data variability, indicating less consistency in the model's predictions. The fearful-avoidant cluster occupies an intermediate position, demonstrating predictive accuracy comparable to the Secure group.

## 5 Discussion

In recent years, there has been increasing evidence in the literature that the notion of attachment style is not only an explanatory behavioral construct but also has a profound neurophysiological basis. In that context, the ability of EEG to record rapid temporal signals evoked by emotional responses to success or failure signals provides a breeding ground for researching the neuro correlates of attachment style.

The results presented in this paper can be summarized into three main insights. First, and the most important one is a demonstration of the ability to detect attachment style based solely on EEG data gathered from a general cognitive task, the Flanker task, thus cannot be manipulated by the participants. Second, there is a dependency between both attachment dimensions, that is, anxiety and avoidance. Third, features extracted from EEG signals on a task that has no direct relation to the notion of attachment can be used to construct a predictive model for attachment styles.

Unlike our previous EEG work, which focused on binary classification between secure and insecure attachment styles, the present model advances the field by predicting continuous anxiety and avoidance scores. While this dimensional approach enables approximate *post hoc* mapping onto the four canonical attachment styles, its primary strength lies in modeling the spectrum of individual differences across both axes. The ability to recover these scores from neural data without relying on self-report represents a meaningful step toward more objective measures of personality-related constructs.

Besides our main results, our research bears additional implications for attachment theory. The significance within the context of the dimensional theory of attachment styles is first to the discussion on whether adult attachment styles are categorical or dimensional (Fraley et al., 2015). First, our results with the multi-target regression model suggest a dependency between both attachment dimensions. In addition, our results, as shown in Figure 7, visually underscores the variability in predicting Anxious and Avoidant attachment styles, indicative of broader error ranges in more complex attachment dimensions. These findings support the dimensional perspective of attachment theory, emphasizing that attachment styles span multiple dimensions, enriching our understanding of human attachment and relationships.

Specifically, the Secure cluster exhibits the tightest grouping, indicating the model's exceptional performance in predicting this attachment style. Conversely, the Anxious and Avoidant clusters, with larger radii, demonstrate varying prediction errors and data variability, suggesting less consistency and precision. The Fearful Avoidant cluster falls in between, indicating comparable predictive accuracy to the Secure group. These findings harmonize with established literature that posits that Secure and Fearful Avoidant attachment occupy opposite ends of the spectrum of attachment styles (Brennan et al., 1998). This highlights attachment styles' complex and continuous nature, supporting the dimensional approach within attachment theory.

Our study also provides insights for AI researchers with complex, dimensional data. The ability to successfully construct a predictive model relies not on technological advancement in EEG technology but on machine learning technologies and processing power improvements. To make our model, we present a practical example of using ROCKET for time-series data feature extraction, paired with CatBoost for analysis. Specifically, the ability to automatically create a dataset of 20,000 features and reduce them using PCA to a subset of 87 features is due to newly developed algorithms unavailable a few years ago.

Thus, beyond theoretical contributions, our study also diverges methodologically from traditional EEG-based attachment research, which has largely relied on predefined features such as frontal alpha asymmetry (Rognoni et al., 2008) or ERP components (Zhang et al., 2023). These hand-crafted measures restrict analysis to a narrow set of expected neural markers. In contrast, the ROCKET algorithm enables automated extraction of a broad range of time-series features directly from the EEG signal, without relying on prior assumptions. This data-driven strategy allows for a more expansive and potentially more sensitive representation of neural dynamics associated with attachment, making use of computational tools that only recently became viable.

Beyond classification accuracy, our approach offers several advantages over traditional self-report measures of attachment. First, EEG-based measures avoid the subjectivity and social desirability biases that can distort questionnaire responses and reduce their validity (Van de Mortel, 2008). Second, EEG records implicit neural responses in real time, which are not limited by self-awareness or language, and in some cases have been shown to predict behavior more directly than questionnaires (Shestyuk et al., 2019). Third, EEG is non-invasive, portable, and suitable for assessment outside the laboratory, which increases flexibility and ecological validity (Sugden et al., 2023). In the present study, self-reported attachment scores served as reference labels to demonstrate that EEG signals contain patterns consistent with these dimensions. While this means the model is anchored to self-reports here, the approach lays the groundwork for using EEG as an independent or complementary tool, particularly in situations where self-report is unreliable or not possible.

Our exploratory study is not without limitations, and the following list provides some of them and ideas for future studies. Addressing these limitations and incorporating the proposed future directions could improve the understanding and prediction of attachment styles. This progress may benefit psychological practices and contribute to more tailored therapeutic approaches.

1. Limited Sample Size: Our study's sample size, especially within the secure attachment group, was limited due to the proportional allocation method. Future studies should strive for more extensive and varied sample sizes to ensure broader applicability of the results.
2. Homogeneity of Participants: We concentrated on university students, potentially limiting the broader applicability of our findings. Future work should seek to incorporate a demographically wider participant cohort with varying ages, cultural contexts, and life experiences to bolster the universality of the results.
3. Engineering Enhancements: Technologically, it would be advantageous for subsequent studies to investigate the creation of real-time EEG data processing systems to predict attachment styles. Such systems could prove invaluable in clinical settings for immediate interventions. Improving the model's computational efficiency for use on devices with limited resources would also facilitate wider deployment and access.
4. Longitudinal Analysis: Incorporating a longitudinal study design could provide insights into the stability of attachment styles over time and the model's predictive power across different life stages.
5. Interdisciplinary Approaches: Combining psychological theory with advanced machine learning techniques can be further explored. For example, integrating neurobiology and psychodynamic theory findings might refine the model's predictive capabilities.
6. Cross-Validation with Behavioral Data: To enhance the validity of our model, future studies could incorporate behavioral measures of attachment, providing a multimodal approach to validation and capturing a more holistic picture of attachment patterns.
7. Feedback Type Separation**:** EEG epochs were pooled across all feedback types (positive and negative) rather than analyzed separately. While this approach aligned with our aim of modeling general post-feedback neural responses, it does not allow us to isolate neural mechanisms specific to success vs. failure outcomes. Future research could extend the current work by examining condition-specific neural responses, an approach we have implemented in related work examining the interplay between feedback type, task difficulty, and attachment style (Mizrahi et al., 2025).

## 6 Conclusions

Attachment style is one of the most essential psychological constructs that dictates human behavior in their younger years and adult life. While there have been insights through previous studies showing different neurophysiological evidence of attachment, there has yet to be a successful attempt at predicting attachment style based on neurophysiological signals in recent years. The current research fills this gap by demonstrating that a predictive regression model can be constructed on EEG data in a way that allows for the prediction of one's attachment style through anxiety and avoidance dimensions with high accuracy.

The implications of the study are profound. First, in contrast to the available ways of measuring attachment via self-report questionnaires, our method does not reveal what is being measured by the participant. As such, our suggested method tackles one of the main limitations of self-report evaluation, i.e., the inherent bias of the participant. Second, we show for the first time that instead of relying on common EEG features from the literature (ERP, alpha, beta, gamma values, etc.), one could use the newly available machine learning algorithm, ROCKET to automatically construct a large set of features, reduce it to its prominent vector via a PCA algorithm, and use those as the input to the prediction model. This new technique presented in this paper allows for the construction of a successful prediction model even in cases where the physiological understanding of the brain signals and their relation to the object in question (in our case attachment style) is still a mystery.

We believe the technique demonstrated here could be easily replicated and utilized to learn and predict other cognitive constructs or personality traits such as the Big Five traits, approach vs. avoidance motivation, and many others that are still measured using self-report questionnaires. In contrast to prior approaches relying on explicit feature extraction across known signal domains followed by filter-based selection algorithms (e.g., Relief) (Bunterngchit et al., 2024), our method applies automated convolutional transformations (ROCKET) to derive a large feature set without predefined signal assumptions. More broadly, this work shows how newer machine learning tools for time-series data can help uncover psychological patterns in brain activity, even without relying solely on predefined EEG markers or manual feature engineering. We hope this encourages future research to apply similar approaches in the search for neural markers of personality and cognition in ways that complement existing psychological theory.

## Data Availability

The raw data supporting the conclusions of this article will be made available by the authors, without undue reservation.
